# A Case of Scrotal Haematocele

**Published:** 1908-06

**Authors:** Whatley W. Battey

**Affiliations:** Assistant Professor, Anatomy and Clinical Surgery, Medical Department, University of Georgia


### A CAUSE OF SCROTAL HAEMATOCELE.*

BY WHATLEY W. BATTEY, JR., M. D., ASSISTANT PROFESSOR, ANATOMY AND CLINICAL SURGERY, MEDICAL DEPARTMENT, UNIVERSITY OF GEORGIA.

The tunica vaginalis testis is the location of fluid accumulation produced by itself the lining membrane possessing that property, or by injury of the testicle there being a gradual outpouring into its cavity, further gonorrhoeal inflammation of epididymis syphilis and new growths act likewise. This hydrocele fluid may become discolored being in part due to its age, becoming dark yellow or brown. The gradual accumulation of this fluid does not produce symptoms alarming to patient, until he actually has pain and marked discomfort. When such a condition presents itself he then seeks relief, not always to the physician who is competent to make a diagnosis and suggest appropriate treatment, but to the druggist who at a glance makes a diagnosis of hernia; gives him a suspensory or applies a truss, and leaves the sufferer in as deplorable a condition as before increasing the liability to complications. Imagine a patient with a small hydrocele and varicocele harnessed up in a truss. The direct effect being not only uncomfortable, but adding "fuel to the flame," so to speak, by the pressure of truss against spermatic cord as it emerges from external ring, thus interfering with venous return circulation, and constantly favoring increased varicosities. The pressure produced within tunica vaginalis testis is equal on all sides and as compression of brain is produced, according to some authorities by a rupture of small twigs of blood vessels due to cerebral fluid being set in motion, so will fluid collections in scrotum under influence of traumatism act upon contents of scrotum producing a rupture of circulatory apparatus, provided that varicosities exist, or a simple exudation of secrum as the direct effect of impaction, constituting the condition known as haematocele. Among other causes may be enumerated the gradual thickening of tunica vaginalis, and lastly repeated punctures of hydrocele.

*Read before the Georgia Medical Association, Fitzgerald, April 15-16-17, 1908.

### SYMPTOMS.

The chief symptoms are pain, swelling of scrotum, blood extravasation.

### DIAGNOSIS.

This is not always easy. The history of the case will give important diagnostic data. This condition may be confounded wich hydrocele and hernia. The important diagnostic points in favor of each are as follows:

*Hydrocele.*

Gradual increase in size. Translucency, presence of fluctuation. Pyriform in appearance. Closure of external ring. Absence of impulse upon coughing. Non-reducibility. Absence of inflammation or pain.

*Hernia.*

Doughy feel of scrotum. Impulse upon coughing at external ring. Enlargement of external ring. Posibility of reduction. Presence of marked discomfort. Occasional tympany in other than epiplocele.

*Haematocele.*

Presence of ecchymosis. History of traumatism. Absence of indications of strangulation. History of the previous existing hydrocele.

To show how important a factor traumatism is in the production of this trouble, I can best do so by reporting a case in point.

Mr. G., a carpenter, age 48, noticed two years ago, a swelling of right side of scrotum. This was accompanied by dragging sensation in testicle, partially incapacitating him from work. He applied for aid at a drug store, and he was given a neatly fitting truss, the druggist making a diagnosis of hernia. This truss was worn constantly for two years. On November 19th, 1907, while at work lifting lumber, in an attempt to stoop, the patient had sharp pain in right side of scrotum, which became more intense, and he was sent home, being unable to resume his duties. I saw him the following day. Upon examination of scrotum noted the following condition: Right scrotum very much enlarged and ecchymotic, owing to extravasated blood. There was swelling in the inguinal region just to the outside of external ring, which was painful upon pressure, resembling hernial protrusion. With the tumor in inguinal region, and enlargement of scrotum, and the history of patient having worn a truss, I at once concluded that the case was one of incarcerated hernia, and probably strangulated. The general condition of patient was good. Bowels had not been moved since the day before, but abdomen was soft and not painful. There was no nausea or vomoting. I advised an operation, which advice was accepted. Under ether narcosis, made an incision over tumor in inguinal region down to sac. Upon opening sac expecting to liberate intestine or omentum, I was surprised to find accumulated serosanguinous fluid and blood clots, the sac communicating with scrotum and not with peritoneal cavity. Upon further exploration found rupture of a varicose vein of cord, the hemorrhage from same having been arrested by pressure within sac. Cavity of tunica vaginalis was thoroughly cleansed out and same closed by suture above and Volkman operation done on scrotum. Serosanguinous fluid drained away for ten days, when incision finally healed, despite efforts to keep it open. Patient left hospital with slight swelling of tunica vaginalis testis with the direction to return and have scrotum operated upon later, for hydrocele under cocaine anaesthesia, using aspiration method.

This case proves beyond a doubt, that we can add to the list of causes of haematocele, rupture of a varicocele and that rupture is more likely to occur in the presence of a hydrocele where the effect of traumatism, owing to pressure from hydrocele, would be greater; that the application of trusses by druggists to patients who do not suffer with hernia should meet with our condemnation.

*Report of a Case of Ingestion of Open Safety Pin by a Child Two Years of Age, Subsequently Passed by Bowels Without Symptoms. By W. W. Battey, Jr., M. D.*

I wish to report this case to show how quickly foreign bodies pass from stomach into bowel, and to show how we may be mislead notwithstanding most perfect skiagraphic charts, and further the absence of pain during passage of foreign body.

W. H., age two and one-half years, while mother was applying a napkin, grabbed safety pin from mother's dress, the same having been placed there while napkin had been previously removed; placing same in mouth, quickly swallowed it. The mother made an attempt to get pin from mouth of child, but efforts were fruitless. The child cried and was strangled considerably as pin passed down the oesophagus. The pin was ingested with circular spring part of pin passing down first. I saw the child fifteen minutes after ingestion. The mother was positive that the child had swallowed pin, but to look at the child one would think that she was mistaken. There was no pain over stomach upon palpation. Water was taken and swallowed without difficulty. I advised a skiagraphic examination of stomach, oesophagus and intestinal tract, which was made by Miss Dendy.

Child was put upon farinaceous foods with directions to watch him carefully for any symptoms of pain or other trouble.

The skiagraph showed nothing in the oesophagus, stomach or intestinal tract. I was inclined to believe that the mother was mistaken. On second morning following ingestion I was phoned that the child had passed the pin.

